# A Web-Based, Positive Emotion Skills Intervention for Enhancing Posttreatment Psychological Well-Being in Young Adult Cancer Survivors (EMPOWER): Protocol for a Single-Arm Feasibility Trial

**DOI:** 10.2196/17078

**Published:** 2020-05-28

**Authors:** John M Salsman, Laurie E McLouth, Michael Cohn, Janet A Tooze, Mia Sorkin, Judith T Moskowitz

**Affiliations:** 1 Department of Social Sciences and Health Policy Wake Forest Baptist Comprehensive Cancer Center Wake Forest School of Medicine Winston Salem, NC United States; 2 Department of Behavioral Science Markey Cancer Center University of Kentucky College of Medicine Lexington, KY United States; 3 Osher Center for Integrative Medicine University of California San Francisco San Francisco, CA United States; 4 Department of Biostatistics and Data Science Wake Forest Baptist Comprehensive Cancer Center Wake Forest School of Medicine Winston Salem, NC United States; 5 Department of Obstetrics & Gynecology University of Chicago Chicago, IL United States; 6 Department of Medical Social Sciences Northwestern University Feinberg School of Medicine Chicago, IL United States

**Keywords:** emotions, telemedicine, happiness, eHealth, cancer, young adult, internet, mobile phone

## Abstract

**Background:**

Adolescent and young adult cancer survivors (AYAs) experience clinically significant distress and have limited access to supportive care services. Interventions to enhance psychological well-being have improved positive affect and reduced depression in clinical and healthy populations but have not been routinely tested in AYAs.

**Objective:**

The aim of this protocol is to (1) test the feasibility and acceptability of a Web-based positive emotion skills intervention for posttreatment AYAs called Enhancing Management of Psychological Outcomes With Emotion Regulation (EMPOWER) and (2) examine proof of concept for reducing psychological distress and enhancing psychological well-being.

**Methods:**

The intervention development and testing are taking place in 3 phases. In phase 1, we adapted the content of an existing, Web-based positive emotion intervention so that it would be suitable for AYAs. EMPOWER targets 8 skills (noticing positive events, capitalizing, gratitude, mindfulness, positive reappraisal, goal setting, personal strengths, and acts of kindness) and is delivered remotely as a 5-week, Web-based intervention. Phase 2 consisted of a pilot test of EMPOWER in a single-arm trial to evaluate feasibility, acceptability, retention, and adherence and to collect data on psychosocial outcomes for proof of concept. In phase 3, we are refining study procedures and conducting a second pilot test.

**Results:**

The project was part of a career development award. Pilot work began in June 2015, and data collection was completed in March 2019. The analysis is ongoing, and results will be submitted for publication by May 2020.

**Conclusions:**

If this intervention proves feasible and acceptable, EMPOWER will be primed for a subsequent large, multisite randomized controlled trial. As a scalable intervention, it will be ideally suited for AYA survivors who would otherwise not have access to supportive care interventions to help manage posttreatment distress and enhance well-being.

**Trial Registration:**

ClinicalTrials.gov NCT02832154, https://clinicaltrials.gov/ct2/show/NCT02832154.

**International Registered Report Identifier (IRRID):**

DERR1-10.2196/17078

## Introduction

### Background

Adolescent and young adult cancer survivors (AYAs) are an important underserved group at risk for significant psychological distress. There are approximately 70,000 new diagnoses of cancer annually in AYAs (aged 18-39 years) [1]. Currently, nearly 2 million people in the United States are living with or have survived being diagnosed with cancer as an AYA. Five-year survival rates of AYAs are high (>80%) [2], and AYAs have approximately 35 to 59 years of life expectancy remaining [3], underscoring the importance of posttreatment survivorship care. AYAs face unique challenges, given the physical, cognitive, and psychosocial developmental milestones disrupted as a result of cancer [4,5]. Notably, the prevalence of clinically significant depression or anxiety is much higher compared with older adults [6-12]. For older adults, cancer is a distressing event but a more normative experience in an aging population. In addition, older adults typically have greater experience in coping with major life events. For AYAs, a cancer diagnosis is routinely unexpected, considerably disruptive, and frequently socially isolating, factors that contribute to higher rates of psychological distress. Moreover, AYAs may have inadequate insurance coverage, limited financial assets, and experience significant work interruption, leading to greater financial strain and contributing to elevated distress [13,14]. Accordingly, AYAs can benefit from targeted, supportive care interventions to decrease distress and enhance well-being as they navigate posttreatment survivorship.

The National Cancer Institute has called for supportive care interventions in AYAs to address psychological health deficits [15]. Although a modest but growing number of psychosocial interventions have been developed for AYAs [16,17], including those that use electronic health (eHealth) modalities [18-20], none have included a focus on enhancing psychological well-being through positive emotions. eHealth interventions represent promising options for patient engagement, especially with *digital natives* such as AYAs, and provide opportunities for fostering user engagement, which is positively associated with intervention efficacy [21]. The vast majority of AYAs access the internet (94%-99%) [22] and own smartphones (92%-96%) [23]. As AYAs have shown that they prefer remotely delivered, on-demand interventions [24], there is a clear need and opportunity for eHealth interventions to positively impact AYAs’ psychological well-being. Moreover, although the deleterious effects of psychological distress are well researched, comparatively less attention has been focused on the benefits of psychological well-being. Psychological well-being is significantly associated with better health outcomes (better physical health [25] and lower risk of mortality in healthy and chronically ill samples [26-30]), is unique from the influence of distress, and includes domains that are inherently valued by patients (better relationships, more creativity, and better work quality [31]).

### Objectives

In this protocol paper, we describe the development and pilot testing of a Web-based positive emotion skills intervention for posttreatment AYAs, Enhancing Management of Psychological Outcomes With Emotion Regulation (EMPOWER). We are adapting an existing multicomponent positive emotion skills intervention [32-36] and tailoring it for AYAs. EMPOWER is a 5-session intervention designed to teach participants 8 skills for increasing positive emotion in their daily lives.

The objectives of this investigation are to (1) test the feasibility and acceptability of a Web-based positive emotion skills intervention tailored for AYAs posttreatment and (2) examine proof of concept of the positive emotion skills intervention for reducing psychological distress (depression, anxiety, and anger) and enhancing psychological well-being (positive affect, life satisfaction, meaning and purpose, and general self-efficacy). In addition, exploratory analysis will examine associations with other indicators of health-related quality of life (HRQOL; fatigue, pain interference, sleep disturbance, physical functioning, and social functioning) and health behaviors (diet, exercise, alcohol use, and smoking). Ultimately, this research seeks to develop an optimized Web-based positive emotion skills intervention for posttreatment AYAs, which will be tested in a future randomized controlled trial (RCT).

## Methods

### Overview

The intervention development and testing were planned for 3 phases. Phase 1 aimed to adapt a Web-based positive emotion skills intervention to maximize the acceptability and relevance of the intervention content for posttreatment AYAs. Phase 2 aimed to conduct a pilot test of EMPOWER in a single-arm trial to evaluate feasibility, acceptability, retention, adherence, and collect data on psychosocial outcomes for proof of concept. In phase 3, we incorporate any suggested modifications from the phase 2 pilot to address any potential challenges encountered from the first round of pilot testing and to ensure that we are maximizing our ability to recruit, retain, and support AYAs. These changes are followed by a second round of pilot testing. Planned accrual was 20 for phase 2 and 20 for phase 3.

Participants were recruited through 2 comprehensive cancer centers (the Robert H Lurie Comprehensive Cancer Center [RHLCCC], and the Wake Forest Baptist Comprehensive Cancer Center [WFBCCC]) and supplemented by recruitment over social media. All participants were asked to provide daily emotion reports over the course of the 5-week intervention and received self-paced Web-based instruction and practice in skills for increasing their daily experience of positive emotion. Participants were assessed at baseline, at 8 weeks (immediately postintervention), and at 12 weeks. To minimize participant burden, we used brief and well-validated National Institutes of Health (NIH) Patient-Reported Outcomes Measurement Information System (PROMIS) and NIH Toolbox measures to assess most study outcomes.

### Phase 1: Intervention Adaptation

As the first step in this phase, the study principal investigator (PI: JS) reviewed candidate interventions for potential adaptation and testing among AYA posttreatment survivors. The MARIGOLD intervention, developed by a lead collaborator (JM) for individuals with elevated depressive symptoms, provided the constellation of skills to promote positive emotions through emotion regulation and was tailored for Web-based delivery [35,36]. MARIGOLD is a 5-session intervention that teaches participants 8 empirically-based skills (ie, positive events, savoring, gratitude, attainable goals, mindfulness, positive reappraisal, personal strengths, and acts of kindness) to increase the frequency of positive emotions experienced in their lives. As AYAs are digital natives, having access to and comfort with digital technologies [22,23], this mode of intervention delivery was well suited for them.

In the second step of this process, the study team reviewed the intervention content with a particular focus on ensuring that the appropriate coping skills were represented, and the language used was applicable for posttreatment AYAs. As a third step in this process, we solicited stakeholder input from AYAs and their providers. Stakeholders reviewed the intervention content and provided feedback on the quality of advice (eg, *Does this sound like something you can do*?), their affective response (eg, *Talk about how reading it made you feel*.), and the appropriateness of images used in the lessons (eg, *Some pages have a photo or video. Give your comments on that*.). All feedback was reviewed and discussed by the full study team to finalize the intervention content before pilot testing.

### Phase 2: Initial Pilot Testing

#### Study Population

Participant eligibility inclusion criteria included (1) able to read and understand English, (2) able to provide informed consent, (3) past history of a cancer diagnosis (excluding basal cell skin carcinoma), (4) 18 to 39 years of age at diagnosis, (5) currently within 0 to 5 years post active treatment, and (6) wireless internet connection or a home computer that is connected to the internet. Exclusion criteria included (1) evidence of cancer recurrence or a history of multiple primary cancers, (2) currently receiving palliative or hospice care, or (3) a significant psychiatric history. Our past work with posttreatment AYAs underscores the psychologically vulnerable posttreatment, *reentry* period, as they navigate new and sometimes recurring challenges to their psychological well-being [6,37-39]. Providing a Web-based, self-guided, well-being intervention during this critical transition phase helps address some of these unmet needs.

#### Study Procedures

##### Recruitment and Enrollment

With prior approval from the medical oncologists, study staff identified potential RHLCCC and WFBCCC patients from the electronic medical record. Potentially eligible patients were recruited through a direct in-clinic approach and mailed letters, followed by a phone call from a study team member. The recruitment call was followed by an email outlining the details discussed during the phone call and instructions on the next steps and a link to the screening questionnaire. The patients were then screened for eligibility using Qualtrics, a Web-based data collection tool that enables researchers to create study-specific websites for capturing participant data securely. Those who were ineligible were shown a message thanking them for their interest but informing them that they were not eligible for the study. Patients who were eligible were navigated to the consent form and initial study questionnaire on Qualtrics. On completion of the baseline questionnaire, all participants were asked to begin daily emotion reporting for 2 weeks before beginning the intervention.

##### Intervention Content

The EMPOWER intervention is a 5-session Web-based intervention that teaches 8 skills for increasing the frequency of positive emotions: (1) *noting daily positive events* [40-43], (2) *capitalizing* on or savoring positive events [44,45], (3) *gratitude* [46-48], (4) *mindfulness* [49-52], (5) *positive reappraisal* [53-58], (6) focusing on *personal strengths* [59-61], (7) setting and working toward *attainable goals* [57,58,62-64], and (8) small *acts of kindness* [65-69] (see Table 1). The skills are presented over 5 weeks. A week consists of 1 to 2 days of didactic material and several days of brief, real-life skills practice and reporting, with each day’s *home practice* taking approximately 20 to 30 min to complete. Participants cannot skip ahead, but they can return to old lessons or exercises if they choose. Most exercises are in *diary* format in which participants’ past responses are displayed next to their new ones so that every time the participant visits that exercise, they see their growing list of past positive experiences. All aspects of the intervention, including the didactics and skills practice, are self-guided and interactive. Additional details of the development of the intervention are published elsewhere [35,36].

**Table 1:** Overview of the skills and content of the Enhancing Management of Psychological Outcomes With Emotion Regulation intervention.

Session and skills		Session content
**Week 1**		
	Positive events	Learning to recognize positive events (eg, a good conversation with a friend, a good cup of coffee) and the associated positive affect.
	Capitalizing	Practicing ways to amplify the experience of positive events (eg, taking an extra moment to savor the experience as it is happening, reliving the positive experience, telling someone else about the positive experience).
	Gratitude	Taking a moment to feel thankful or appreciative of the things you have in life (eg, family, a sunny day, a good night’s rest).
**Week 2**		
	Mindfulness	Learn and practice the awareness and nonjudgment components of mindfulness.
**Week 3**		
	Positive reappraisal	Understanding positive reappraisal and the idea that different forms of positive reappraisal can all lead to increased positive affect in the face of stress (eg, seeing the *silver lining*, finding out things were not as bad as they could have been, identifying good things that came out of the event).
**Week 4**		
	Personal strengths	Participant lists his or her personal strengths and notes how they may have used these strengths recently (eg, having a good sense of humor, being artistic).
	Achievable goals	Understanding the characteristics of attainable goals and setting some goals for the week.
**Week 5**		
	Acts of kindness	Understanding that small acts of kindness can have a big impact on positive emotions (eg, buying the person behind you in line a cup of coffee).

##### Intervention Platform

Our Web intervention is delivered via a customized website built on Moodle, a courseware platform that is used by schools and universities worldwide. Moodle allows the delivery of text or video instruction as well as interactive activities such as journals and adaptive quizzes. Moodle is recognized as secure and well-tested software, and Health Insurance Portability and Accountability Act-compliant hosting is provided by the Northwestern University. All communications with the website use industry-standard transport layer security or secure sockets layer encryption. Another layer of security is provided by avoiding any use of personally identifiable information, medical information, or other sensitive information in the context of the intervention. Participants’ Moodle accounts are not linked to their real name or email address. Email and text message reminders are handled by a smartphone Ecological Momentary Assessment text messaging system that does use the participant’s name and email address, but that cannot be linked to their Moodle account. The design of our intervention website has been refined through a number of iterations based on user testing and feedback from study participants (eg, simplifying the interface and clearly labeling new material and exercises). We have also ensured that material is viewable on handheld, tablet, and laptop devices.

##### Acceptability Interview

Research staff conduct a 30-min audio-recorded, postintervention phone interview with all participants approximately 1 week after the intervention is complete to gather acceptability data. Participants are asked to rank order their favorite intervention skills, their intentions to practice each of the skills, and their plans for continued practice. In addition, they are asked whether or not they would recommend the intervention to a friend or someone newly diagnosed with cancer.

##### Participant Incentives

Each participant is paid US $10 for each completed assessment for a maximum of US $30. In addition, participants are paid US $0.25 for each of three daily emotion assessments over the two separate 2-week reporting periods (4 weeks; 28 possible daily reports, up to US $21 per participant). In total, participants are compensated a maximum of US $51 for their participation in the study and are paid in full on completion of the study via a virtual gift card.

##### Fidelity Monitoring

We record how frequently participants visit the website and how many times they complete the daily practice exercises for each skill. This information can be used in *dose-response* analyses to determine if greater exposure to the exercises leads to stronger intervention effects. We monitor participant progress during the study and contact participants who appear to be having trouble or disengaging from the intervention. Our experience indicates that even very brief human contact can increase participants’ commitment to the intervention. Participants receive an email or phone call from a study staff member if they fail to visit the website for more than 3 days in a week. Participants who cannot be reached or who do not resume visiting the website but also do not ask to leave the study are recontacted once per week for 3 weeks. After that time, they are counted as noncompleters, although we still try to contact them to obtain postintervention measures. Participants who do not reach the final lesson at the end of 10 weeks are also considered noncompleters and asked to take the postintervention measures at that time.

#### Measures

Patients complete self-report questionnaires throughout the intervention designed to evaluate state and mood-based aspects of psychological well-being as well as related patient-reported outcomes that may be impacted (ie, HRQOL and health behaviors) as a result of changes in psychological well-being. Psychological well-being includes both negative and positive aspects and is assessed by daily emotion reports (ie, run-in period before the intervention, end of day recall during the intervention, and run-out period after the intervention) and by weekly recall measures at baseline (pretest), approximately 8 weeks after baseline (posttest), then at 12 weeks (follow-up). The HRQOL and health behavior measures are also administered at baseline/pretest, posttest, and then follow-up (see Multimedia Appendix 1). All measures are completed from home via participants’ PCs. In addition to the measures listed below, we assess key demographics (race/ethnicity, education, household income, and insurance status), cancer type, time since diagnosis, type of treatment, and time since treatment.

#### Daily Emotion Reports

Daily frequency of positive and negative affect is assessed using modified versions of the NIH Toolbox positive affect short form [70] and the NIH PROMIS depression and anxiety short forms [71]. Participants are asked to respond to each item in terms of how they feel *today*. During the 2-week *run-in*/*run-out* period (weeks 1 and 2 and weeks 11 and 12), all participants complete the daily emotion reports 3 times per day with respect to their emotions at that moment. The purpose of the run-in period is to address any technical issues that participants experience, to ensure participants are comfortable reporting their emotions, to evaluate compliance with completing these reports, and to provide a pre- and postcomparison of state-based affective experiences. Furthermore, the study is designed with a relatively intensive engagement process, and we sought to identify participants who were willing and able to comply with the modest but frequent assessments, didactics, and skills practice that are part of EMPOWER. If participants do not complete at least nine daily reports in a week’s time, they are excluded from further participation in the study. In this circumstance, the participant is notified by email. One week before the 12-week assessment point, participants are contacted and asked to provide the last 2 weeks of daily emotion reporting in time to complete the final assessment. During the 5-week intervention, participants complete the end of day recall at the end of each day with respect to their emotions that day.

#### Psychological Well-Being

Psychological well-being is assessed with NIH Toolbox short forms, capturing 3 common components: positive affect, life satisfaction, and meaning and purpose [70]. In addition, the NIH PROMIS general self-efficacy short form [72] is administered, as it is a closely related construct to psychological well-being and positively associated with better health-related outcomes.

#### Health-Related Quality of Life

We use the PROMIS global health items to assess overall HRQOL [73] and the PROMIS-29 [74,75] to assess domain-specific aspects of HRQOL. The PROMIS global scale consists of 10 items that assess general health, including overall physical and mental health. The PROMIS-29 consists of 29 items that assess physical functioning, anxiety, depression, fatigue, sleep disturbance, social functioning, pain interference, and pain intensity. These PROMIS measures are supplemented with additional items from the PROMIS physical function short form [76] and the PROMIS anger short form [71]. These measures were included to identify potential *signal relationships* for psychological well-being and HRQOL.

#### Health Behaviors

Healthy behaviors often associated with enhanced coping and better psychological adjustment are assessed [77]. Physical health behaviors include diet [78], exercise [79], alcohol consumption [78], and cigarette smoking [78].

### Phase 3: Subsequent Pilot Testing

Primary outcomes will be reviewed and evaluated by the study team. If any outcomes are suboptimal (poor adherence, retention, and accrual), modifications to study procedures will be discussed by the team and implemented to attempt to improve these primary outcomes. A second round of pilot testing will then be conducted to evaluate the same primary and secondary outcomes with a new sample of AYA survivors. Study population, measures, and analytic plans are expected to remain largely unchanged.

#### Analysis Plan

##### Analysis of Primary Objectives

Accrual will be estimated as the number of patients accrued divided by the number of months of accrual. A 95% CI for the monthly accrual rate will be calculated based on the Poisson distribution. The refusal rate will be estimated as the number of patients who refuse to participate divided by the number eligible. Retention will be primarily defined as the proportion of patients who provide 8-week and 12-week data. Patients who discontinue the intervention (refuse phone calls) but complete the outcome assessments will be counted in the numerator for calculating retention. Retention estimates will be calculated overall and by site. Adherence to the intervention will be calculated as the number of intervention sessions completed, the frequency of completing exercises, and the number of website visits. We will calculate and report the mean adherence across all individuals as well as the proportion of patients who completed 3 or more sessions. Several measures will be used to quantify acceptability, including quantitative measures and interviews. Means and the proportion responding affirmatively to the highest 2 responses for each question will be combined, and exact 95% CIs will be calculated for these estimates.

##### Analysis of Secondary Objective

Quantitative outcomes will be assessed by a covariance pattern model for repeated measures to examine the change in patient-reported outcomes over time.

##### Power and Sample Size

Although this is a pilot study, and we will not be testing the efficacy of the intervention, we want to estimate feasibility, acceptability, and changes in patient-reported outcomes with a fair degree of precision. With a total of 40 patients, we can estimate CIs around means within SD 0.31 and proportions within SD 15.5%, with 95% CI. Assuming 20% of the patients may drop out, we could estimate CIs for means within SD 0.35 and proportions within SD 17.3% for measures evaluated at the end of the study.

## Results

### Phase 1: Intervention Adaptation

The project was part of a career development award, funded in September 2011, and the pilot work began in June 2015 with intervention adaptation efforts. We first reviewed the MARIGOLD Web-based protocol in detail, and skills that were too narrowly focused on the protocol’s prior target of treating depression were removed (ie, behavioral activation). The skills sequence remained the same with the exception of mindfulness, which was substituted for behavioral activation in week 2. Next, the study team reviewed the content language of the intervention and changed terms or phrases to reflect the experiences of having had cancer. For example, content language for the skill of positive reappraisal was changed to reflect commonly experienced feelings and cognitions of cancer survivors. Finally, 4 AYA stakeholders (a pediatric oncologist and AYA Medical Director, a clinical psychologist and Director of AYA Oncology, and 2 posttreatment AYA survivors) reviewed the EMPOWER intervention and provided feedback. All stakeholder input was reviewed and discussed by the study team, and minor modifications were made to content language (eg, adding *fear* as a commonly experienced unpleasant emotion among cancer survivors) and images (eg, substituting an image in the Strengths lesson for one that is more broadly applicable to cancer survivors who may have physical limitations) to finalize the intervention before pilot testing.

### Phases 2 and 3: Pilot Testing

Recruitment began for phase 2 in October 2015, and recruitment began for phase 3 in April 2017. Data collection was completed in March 2019. Data analysis is currently ongoing, and the first results are expected to be submitted for publication in May 2020.

## Discussion

### Principal Findings

This paper describes the study protocol for adapting and pilot testing the EMPOWER intervention, a Web-based positive emotion skills intervention for AYA cancer survivors. In this study, we are tailoring an existing positive emotions intervention to align with the needs and preferences of posttreatment AYAs and then piloting the intervention over two waves of data collection to refine study procedures. Our short-term goal for this work is to produce a multicomponent, emotion regulation intervention that is feasible and acceptable to AYA cancer survivors for future testing as part of a larger RCT.

### Strengths and Limitations

There are a number of strengths to this research study. First, psychosocial interventions to promote psychological well-being are infrequently tested in cancer survivorship despite their potential beneficial effects. In a meta-analysis of interventions that impact well-being outcomes in cancer, 28 RCTs with positive affect outcomes were identified, yielding an overall increase in positive affect (*g*=0.35) [80]. However, only 36% (10/28) of those RCTs were specifically designed to target positive affect, and only 11% (3/28) of those interventions were focused on posttreatment cancer survivors [81-83]. Our dual approach will allow us to impact psychological well-being by reducing and shortening psychological distress as well as increasing and sustaining psychological well-being.

Second, EMPOWER uses a Web-based eHealth strategy that is accessible via desktop PC, tablet PC, or smartphone (both iPhone and Android systems). As already noted, AYAs are *digital natives* and leveraging their technological aptitude for multicomponent, tailored intervention delivery allows us to match their needs and preferences to supportive care content. Moreover, because EMPOWER is scalable, it can be simultaneously delivered to a limitless number of AYAs at multiple and geographically diverse sites. Treatment integrity and fidelity to EMPOWER remain fully intact, reducing threats to internal validity. Thus, there is great long-term potential to reach AYAs who are underserved and might not typically have access to psychosocial services through community-based practices where a majority receive care [84,85].

Third, our approach uses state-of-the-art systems in the measurement of patient-reported outcomes by including emotional, physical, and social health measures from the NIH Toolbox [70,86,87] and NIH PROMIS [88-90]. These psychometrically robust measurement systems have been systematically created through rigorous qualitative and quantitative science methodologies, yielding measures that are reliable, valid, and responsive. Moreover, the static short forms were created by selecting the best performing items that provide coverage to a range of constructs, which helps to minimize respondent burden without sacrificing measurement precision. Thus, we can assess more content-relevant domains with fewer questions.

Despite these strengths, it is worth noting the potential limitations to our work. First, we are conducting a single-arm trial for this pilot study and not randomizing participants to a control arm. Although an RCT is indeed the *gold standard* of intervention research, the single-arm approach is a defensible strategy when examining primary outcomes of feasibility and acceptability for a small pilot study. As part of a future strategy with this research, we are planning to conduct a large RCT. Second, we are not screening participants into the study based on moderate to high distress scores as some emotional well-being interventions typically do. Although such an approach might result in larger effect sizes for our psychological outcomes (both distress and well-being), this would prevent us from exploring the potential benefits of this intervention for those who may not have clinically significant levels of distress but could benefit from improved emotional well-being nonetheless. That said, we are screening out noncompliant participants with our run-in period, and this may result in a selection bias toward a highly motivated and compliant sample. Third, AYA cancer survivors have some of the poorest participation rates in cancer clinical trials (both therapeutic and supportive care) [91-94]. Recruiting AYAs involves significant time and resources. As there is a clear need for interventions that can help improve their psychological well-being, our work is a necessary first step.

Finally, our emphasis on interventions to enhance psychological well-being is not intended to deny, minimize, or otherwise ignore the significant stress of being diagnosed with and treated for cancer as an AYA or the deleterious impact it has on patients’ psychological and physical health. Nor is it advocating a superficial *don’t worry, be happy* approach to dealing with their illness. Rather, we are suggesting that if we broaden our focus to include a wider range of coping strategies, including interventions to promote psychological well-being, we will better equip AYAs to manage the deleterious effects of stress [95].

### Conclusions

The goal of this work is to adapt and pilot test a Web-based, emotion regulation intervention designed to enhance positive emotions among AYA posttreatment cancer survivors. If EMPOWER proves feasible and acceptable, it will be primed for a subsequent large, multisite RCT. As a scalable intervention, it will be ideally suited for AYA survivors who would otherwise not have access to supportive care interventions to help manage posttreatment distress and enhance well-being.

## Appendix

Multimedia Appendix 1 Study timeline.

Multimedia Appendix 2 Peer-reviewer report from NIH.

## Abbreviations

AYA: adolescent and young adult cancer survivor
eHealth: electronic health
EMPOWER: Enhancing Management of Psychological Outcomes With Emotion Regulation
HRQOL: health-related quality of life
NIH: National Institutes of Health
PI: principal investigator
PROMIS: Patient-Reported Outcomes Measurement Information System
RCT: randomized controlled trial
RHLCCC: Robert H Lurie Comprehensive Cancer Center
WFBCCC: Wake Forest Baptist Comprehensive Cancer Center
