# Supplementary material for: A Web-Based, Positive Emotion Skills Intervention for Enhancing Posttreatment Psychological Well-Being in Young Adult Cancer Survivors (EMPOWER): Protocol for a Single-Arm Feasibility Trial
Source: JMIR Res Protoc. 2020 May 28;9(5):e17078. doi: 10.2196/17078 (PMC7290453; doi:10.2196/17078)
Supplement: Multimedia Appendix 1 [file resprot_v9i5e17078_app1.docx]

**Multimedia Appendix 1. Study Timeline**

|  | **Pre-test Questionnaire**  **(Qualtrics)** | **Daily Emotion Reporting (EMA)** | **End-of-Day Recall (EMA)** | **EMPOWER**  **Session 1 (Moodle)** | **EMPOWER**  **Session 2 (Moodle)** | **EMPOWER**  **Session 3 (Moodle)** | **EMPOWER**  **Session 4 (Moodle)** | **EMPOWER**  **Session 5 (Moodle)** | **Phone Interview (via coordinator)** | **Post-test Questionnaire (Qualtrics)** | **Follow-up Questionnaire (Qualtrics)** |
| --- | --- | --- | --- | --- | --- | --- | --- | --- | --- | --- | --- |
| Week 1 | ✓ | ✓ |  |  |  |  |  |  |  |  |  |
| Week 2 |  | ✓ |  |  |  |  |  |  |  |  |  |
| Week 3 |  |  | ✓ | ✓ |  |  |  |  |  |  |  |
| Week 4 |  |  | ✓ |  | ✓ |  |  |  |  |  |  |
| Week 5 |  |  | ✓ |  |  | ✓ |  |  |  |  |  |
| Week 6 |  |  | ✓ |  |  |  | ✓ |  |  |  |  |
| Week 7 |  |  | ✓ |  |  |  |  | ✓ |  |  |  |
| Week 8 |  |  |  |  |  |  |  |  | ✓ | ✓ |  |
| Week 9 |  |  |  |  |  |  |  |  |  |  |  |
| Week 10 |  |  |  |  |  |  |  |  |  |  |  |
| Week 11 |  | ✓ |  |  |  |  |  |  |  |  |  |
| Week 12 |  | ✓ |  |  |  |  |  |  |  |  | ✓ |
