# Supplementary material for: A Web-Based, Positive Emotion Skills Intervention for Enhancing Posttreatment Psychological Well-Being in Young Adult Cancer Survivors (EMPOWER): Protocol for a Single-Arm Feasibility Trial
Source: JMIR Res Protoc. 2020 May 28;9(5):e17078. doi: 10.2196/17078 (PMC7290453; doi:10.2196/17078)
Supplement: Multimedia Appendix 2 [file resprot_v9i5e17078_app2.pdf]

SUMMARY STATEMENT
(Privileged Communication)

PROGRAM CONTACT:
Susan Perkins
301-496-8580
perkinsu@mail.nih.gov

Release Date: **08/10/2011**

Application Number: **1 K07 CA158008-01A1**

Principal Investigator

SALSMAN, JOHN PHD

Applicant Organization: NORTHWESTERN UNIVERSITY

Review Group: **NCI-J**
Subcommittee J - Population and Patient-Oriented Training

Meeting Date: **06/30/2011** ***RFA/PA:*** **PAR09-078**
Council: **OCT 2011** ***PCC:*** **S9TR**
Requested Start: **09/01/2011**

Project Title: **Assessment, Development and Application of Positive Psychology Science for Cancer**
SRG Action: **Impact/Priority Score: 20**
Human Subjects: 44-Human subjects involved - SRG concerns
Animal Subjects: 10-No live vertebrate animals involved for competing appl.
Gender: **1A-Both genders, scientifically acceptable**
Minority: **1A-Minorities and non-minorities, scientifically acceptable**
Children: **1A-Both Children and Adults, scientifically acceptable**
Clinical Research - not NIH-defined Phase III Trial

Project Year	Direct Costs Requested	Estimated Total Cost
1	124,346	133,504
2	127,335	136,713
3	129,791	139,350
4	133,663	143,507
5	136,771	146,844
TOTAL	651,906	699,919

ADMINISTRATIVE BUDGET NOTE: The budget shown is the requested budget and has not been adjusted to reflect any recommendations made by reviewers. If an award is planned, the costs will be calculated by Institute grants management staff based on the recommendations outlined below in the COMMITTEE BUDGET RECOMMENDATIONS section.

RESUME AND SUMMARY OF DISCUSSION: This is a resubmission of an application for a K07 Cancer Prevention, Control, Behavioral and Population Sciences Career Development Award from Dr. John Salsman, a Research Assistant Professor at Northwestern University. Dr. Salsman is an outstanding candidate with excellent training and publication record. This resubmission is highly responsive to previous critiques and significantly improved, although minor weaknesses remain. Prior concerns regarding RCT training and development, mentors' expertise in RCT development, and mentor coordination in terms of meeting frequency have been addressed. The career development plan is comprehensive and the research plan is significant, well organized, and now includes an intervention phase. The mentoring team is outstanding and there is strong institutional support for the project and candidate. Overall, this is an outstanding application and there is confidence that it will lead to independence of the candidate.

DESCRIPTION (provided by applicant): This career development application proposes a mentored training and research experience to develop John Salsman, Ph.D., as an independent investigator in the development of innovative positive psychological outcome measures for cancer prevention and control. Patient-reported outcomes (PROs) capture the patient's unique perspective on the impact of chronic diseases such as cancer and assess subjective experience in ways distinct from physiological outcomes. The Patient-Reported Outcomes Measurement Information System (PROMIS) is an NIH Roadmap initiative designed to improve and standardize PROs for patients with a wide range of chronic diseases and demographic characteristics using state-of-the-art psychometric methods. It is an ambitious and comprehensive PRO measurement system that applies item response theory (IRT) modeling to health status assessment. PROMIS has developed and calibrated item banks assessing emotional distress, sleep-wake function, physical function, pain, fatigue, and social health. There remains a gap in the PROMIS domain framework, with no current banks assessing positive psychological functioning (PPF). As more people survive and even thrive with cancer, understanding positive adjustment and growth in cancer is an important complement to understanding impairment, disability and psychosocial morbidity in cancer prevention and control research. Dr. Salsman proposes (1) to develop new item banks for positive psychological functioning (PPF) to augment and expand the existing PROMIS item banks, (2) to explore the utility of the PPF banks across the cancer care continuum using multiple assessments with a heterogeneous group of cancer patients and survivors, and (3) to adapt and pilot test an intervention to promote well-being among young adult cancer patients and survivors to determine feasibility and acceptability. Results will lead to further refinement of innovative measures of PPF and guide development of future interventions to minimize the adverse impact of cancer and enhance adaptive functioning. The proposed research is supported by a training plan that involves interactions with multidisciplinary colleagues and formal coursework in clinical trials, qualitative methodologies, biostatistics, psychometrics, and positive psychology.

CRITIQUE 1:

Criterion Scores

Candidate:	1
Career Development Plan:	2
Research Plan:	3
Mentor(s), Co-Mentor(s), Consultant(s), Collaborator(s):	1
Environment and Institutional Commitment:	1

Overall Impact: This is a revised application from an outstanding candidate with a very strong mentoring team. There were many strengths noted in the first submission which included a significant area that has received limited attention, an outstanding applicant with excellent training and mentorship in cancer control, good integration into the PROMIS network thus facilitating the research objectives,

and strong evidence of collaboration with the mentoring team. A relatively strong training plan and a feasible research plan with a strong measurement and assessment approach of positive psychological functioning measures were also notable strengths. However, there were multiple weaknesses noted in the first submission that dampened enthusiasm for the application. These included limited training in RCTs despite the development and implementation of RCTs to promote positive psychological functioning as a training goal, a limited research plan that did not involve a pilot of an RCT and lacked in details regarding several components such as the focus groups, several measures and several proposed statistical analyses. There was also some concern raised about the expertise of the mentoring team specific to RCTs. Overall, the applicant has been very responsive to the prior critiques. The career development plan now includes enhanced training components in RCTs that are tied to an added feasibility trial in the research plan, and issues regarding contact time with mentors and expertise in RCTs have been adequately addressed. The research plan now includes an intervention component and details are now provided regarding the statistical analyses. There are several remaining concerns, including lack of a randomized design in the intervention component and concerns over the heterogeneity of the sample in Aim 2 of the research plan. However, these concerns, balanced against the multiple strengths of the application and the responsiveness of the applicant, make this an outstanding application.

SCORED REVIEW CRITERIA

1. Candidate:

Strengths

- Outstanding candidate with excellent pre- and post-doctoral training in cancer control with a programmatic line of inquiry (positive psychology) and outstanding mentorship
- A continued active publication record with 16 peer-reviewed publications, 6 first authored—all relevant to cancer control or positive psychology (3 more publications since last submission and 2 currently under review)
- Significant involvement in NIH funded research as co-Investigator and good evidence of ability to collaborate with other scientists, including proposed primary mentor
- PI on an ACS developmental grant addressing positive adaptation and health behaviors in young adult cancer survivors
- Well integrated into the PROMIS network, should facilitate carrying out the proposed scope of work
- Relevant experience via involvement in several NIH funded projects evaluating outcomes measurements
- Significant scientific service
- Letters of support from scientists not associated with this application are very strong

Weaknesses

- No notable weaknesses

2. Career Development Plan:

Strengths

- Clearly stated training goals which involve acquiring skills in psychometric applications, advanced statistics, biobehavioral assessment and positive psychology—all of which nicely complement the candidate's existing competencies
- Proposed training goals are tied to the stated research goals of developing new item banks for positive psychological functioning (by expanding the PROMIS item banks) and exploring the utility of the positive psychology items on cancer care and adjustment
- Comprehensive training in areas that will complement the candidate's existing competencies (e.g., SEM, cancer epidemiology, qualitative research methods, etc.)
- The proposed master of Applied Positive Psychology is a particular strength of the training plan
- Prior weaknesses involved concerns over the course load associated with the MAPP program, no training plans in grant development and writing, very limited training in development and implementation of RCTs and lack of details regarding the R01 product form this application. Other weaknesses involved concerns over the applicant's need for the proposed training given the absence of training components specific to RCTs.
- The applicant has been very responsive to the concerns raised in the prior reviews. The workload involved in the MAPP has been clarified and seems feasible as the program is developed for working professionals, he has been involved in grant development workshops and plans further training and has also added coursework in clinical trials and a research component to pilot an RCT in PPF. Furthermore, the topic of the R01 that will stem from this application is provided (an RCT evaluating a positive affect intervention to promote adaptation in cancer patients). There also an increase in contact time with the mentoring team.

Weaknesses

- The course in clinical trials takes place in Year 5. This course should take place prior to pilot testing the PPF intervention.

3. Research Plan:

Strengths

- Well organized research plan
- Significant and novel area given the lack of sophisticated assessment of positive psychological functioning despite growing evidence suggesting the utility of this construct
- Research activities are clearly delineated in a timeline with a plans for manuscripts, presentations and an R01 submission
- Prior concerns involved lack of details regarding the focus group in Aim 1 or details for carrying out the psychometric analyses, SEM or mediation analyses, no rationale for the selection of four cancer sites or for targeting anger, poor description of several study measures and no RCT component.
- The applicant has been very responsive as the research plan now involves a pilot test of a RCT to promote PPF, details of the focus group are now provided, as well as more details regarding the CFA and EFA. A rationale for including four cancer sites and including anger is now provided. Similarly, details regarding the statistical analyses specific to the mediation tests and the assessment of health behaviors are now listed in the research plan. A focus of the pilot intervention on colorectal cancer is an added strength.

Weaknesses

- There is some remaining concern over the sample size for Aim 2. The 200 cancer patients and survivors represent a relatively heterogeneous sample in terms of cancer site (4 sites), gender, and stage. Arguably, many of these factors not only impact adaptation to a cancer diagnosis and related treatment, but there are also inherent difference in emotional experiences, expression of affect, responses to interventions, etc. that need to be better addressed.
- It seems that the power analyses for Aim 2 considers the main effects of PFF on physical and mental health independent of possible moderators or covariates.
- The proposed intervention in Aim 3 does not involve an RCT design and while as proposed, this is a logical first step, there remains a missed opportunity to test a small scale feasibility study that involves a randomized design

4. Mentor(s), Co-Mentor(s), Consultant(s), Collaborator(s):

Strengths

- Outstanding mentoring team.
- The primary mentor, Dr. Cella is an outstanding scientist with extensive mentoring experience and given his role in PROMIS, he is ideally suited to mentor this candidate.
- Co-mentors are outstanding as well. Dr. Park is an expert in psychosocial adaptation and positive psychology in cancer survivors. Dr. Hahn has expertise in patient reported outcomes, measurement and advanced statistical methods.
- Letters of support from mentors are very strong, speak highly of the candidate, and show a clear commitment to mentor him with specified contact time with the applicant
- The advisory team is outstanding as well. Expertise and mentoring in IRT is provided by Dr. Choi, while Dr. Clayman will provide expertise in qualitative methods.
- In regard to prior concerns, Dr. Moskowitz, an expert in behavioral interventions to enhance positive affect, has been added to address prior concerns regarding RCT expertise. It has also been clarified that Drs. Cella and Han are both currently conducting RCTs. Concerns over contact time and structure of mentoring meetings has also been adequately addressed.

Weaknesses

- No notable weaknesses.

5. Environment and Institutional Commitment:

Strengths

- Outstanding environment and institutional commitment
- Clear statement from the candidate's department chair (who is also the primary mentor) securing 90% of his time for the proposed scope of work

Weaknesses

- No weaknesses

ADDITIONAL REVIEW CRITERIA (*The following review criteria were not scored individually, but were considered in the determination of the overall impact/priority score*)

Protections for Human Subjects: Acceptable Risks and Adequate Protections

- Adequate steps have been taken to address confidentiality. In the rare event that a participant reports distress over completing measures or participating in the focus groups, adequate steps have been taken for participant referral.

Data and Safety Monitoring Plan: Unacceptable

- No DSMP is in place despite the fact that the research plan involves a behavioral clinical trial that meets the operational definition of a clinical trial per NIH guidelines.

Inclusion of Women, Minorities and Children:

Both Genders, Acceptable

Minority and Non-minority, Acceptable

Children and Adults, Acceptable

- Both genders and minorities are included. Children over the age of 18 are included. Steps have been proposed to assure adequate gender and minority representation

Vertebrate Animals: Not Applicable (No Vertebrate Animals)

Biohazards: Not Applicable (No Biohazards)

Resubmission: The applicant has been very responsive and thoughtful in his response to the prior reviews. The vast majority of concerns have been adequately addressed and have subsequently significantly increased enthusiasm for this application. The remaining concerns, although important, are relatively minor when balanced against the multiple strengths of the application.

ADDITIONAL REVIEW CONSIDERATIONS (*The impact/priority score was not affected by the following considerations*)

Training in the Responsible Conduct of Research: Acceptable

Format: Online CITI training has been completed and additional training modules are proposed. Some coursework also addresses responsible conduct of research.

Subject Matter: Social and Behavioral Science research topics are addressed.

Faculty Participation: Discussions with faculty to address responsible conduct of research is proposed.

Duration: Ongoing CITI training is of acceptable duration.

Frequency: Adequate frequency based on CITI training guidelines.

Select Agents: Not Applicable (No Select Agents)

Resource Sharing Plans: Not Applicable (No Relevant Resources)

Budget and Period of Support: Recommend as Requested

Additional Comments to Applicant: This is an outstanding application by an outstanding candidate with an outstanding mentoring team. As proposed, it will provide the necessary training and development mechanisms necessary for the applicant to develop a successful career trajectory in PPF in cancer control

CRITIQUE 2:

Criterion Scores

Candidate:	1
Career Development Plan:	2
Research Plan:	2
Mentor(s), Co-Mentor(s), Consultant(s), Collaborator(s):	1
Environment and Institutional Commitment:	2

Overall Impact: The candidate presents a revised application focused on developing a positive item bank for the PROMIS system. An intervention to promote positive affect will also be pilot tested. The candidate has a strong publication record. The career development plan includes both formal courses as well as direct mentoring with a strong mentoring team. The research design includes both item development and creation of a pilot intervention. The significance of this proposal stems from the lack of a focus on positive psychology assessment and intervention within the PROMIS system for cancer. There is strong institutional support for the project and candidate. The candidate was responsive to the previous review.

SCORED REVIEW CRITERIA

1. Candidate:

Strengths

- The candidate has a Ph.D. in clinical psychology and completed a post-doctoral fellowship in psycho-social oncology at Northwestern.
- He has been PI on an Illinois Division ACS grant, and is a collaborator on the PROMIS project.
- He has a strong publication record, and is a research assistant professor.

Weaknesses

- No significant weaknesses.

2. Career Development Plan:

Strengths

- The plan includes formal classes as well as individual training experiences.
- There are explicit links between the training and research.

Weaknesses

- Training could be enhanced with further emphasis on interventions.

3. Research Plan:

Strengths

- Measurement and assessment approach is strong.
- Inclusion of an intervention phase.
- This is a well-justified research project.

Weaknesses

- The intervention phase is somewhat under-described, but adds to the impact of the work.

4. Mentor(s), Co-Mentor(s), Consultant(s), Collaborator(s):

Strengths

- A very well-accomplished group, with funding and a history of success in their areas.

Weaknesses

- None.

5. Environment and Institutional Commitment:

Strengths

- Excellent.

Weaknesses

- None.

ADDITIONAL REVIEW CRITERIA (*The following review criteria were not scored individually, but were considered in the determination of the overall impact/priority score*)

Protections for Human Subjects: Acceptable Risks and Adequate Protections

Data and Safety Monitoring Plan: Unacceptable

- No DSMP is included in the application

Inclusion of Women, Minorities and Children:

Both Genders, Acceptable

Minority and Non-minority, Acceptable

Children and Adults, Acceptable

Vertebrate Animals: Not Applicable (No Vertebrate Animals)

Biohazards: Not Applicable (No Biohazards)

Resubmission: This is a responsive resubmission.

ADDITIONAL REVIEW CONSIDERATIONS (*The impact/priority score was not affected by the following considerations*)

Training in the Responsible Conduct of Research: Acceptable

Format: acceptable

Subject Matter: acceptable

Faculty Participation: acceptable

Duration: acceptable

Frequency: acceptable

Select Agents: Not Applicable (No Select Agents)

Resource Sharing Plans: Not Applicable (No Relevant Resources)

Budget and Period of Support: Recommend as Requested

CRITIQUE 3:

Criterion Scores

Candidate:	1
Career Development Plan:	2
Research Plan:	3
Mentor(s), Co-Mentor(s), Consultant(s), Collaborator(s):	1
Environment and Institutional Commitment:	2

Overall Impact: The application presents a comprehensive career development plan and a focused research project that will develop items to assess positive psychological functioning in the PROMIS and explore the utility of these items with cancer patients and survivors. The training plan complements the research by proposing additional training in cancer prevention and control, psychometrics, biostatistics, positive psychology, and behavioral health. This application is presented by an outstanding candidate with an active publication record. Letters of reference and support from proposed advisors were enthusiastic and describe the candidate as well suited for a K07. The candidate was responsive to previous critiques and prior concerns regarding RCT training and development, mentors' expertise in RCT development, and mentor coordination in terms of meeting frequency have been addressed. The mentoring team led by primary mentor Dr. David Cella remains strong and addition of advisor Dr. Judy Moskowitz further strengthens it. This is high impact application from an outstanding candidate and mentoring team.

SCORED REVIEW CRITERIA

1. Candidate:

Strengths

- Candidate committed to a career in cancer prevention and control with strong prior pre-doctoral and post doctoral work experience in the field.
- Candidate has an excellent project management skill set which is needed to accomplish the proposed research.
- Candidate received strong recommendations from independent references that attest to his ability to formulate important research questions and his potential to become an independent researcher.

Weaknesses

- None.

2. Career Development Plan:

Strengths

- Comprehensive training plan.

Weaknesses

- None.

3. Research Plan:

Strengths

- Ambitious research plan that proposes to develop positive psychological functioning item banks for administration with the PROMIS and adaptation of a PPF intervention to determine feasibility.
- Measurement and assessment approaches are strong.
- Candidate is well integrated in the PROMIS network thus facilitating the proposed work.

Weaknesses

- Pilot test for Aim 3: (1) the sample size justification seems appropriate for qualitative analyses but it is unclear as to its suitability for the quantitative outcomes and (2) timeline for aim 3 (1.25 yrs) appears overly long given the goal is to accrue and analyze data from 12 patients.

4. Mentor(s), Co-Mentor(s), Consultant(s), Collaborator(s):

Strengths

- The mentoring team led by primary mentor Dr. David Cella is outstanding
- Addition of advisor Dr. Judy Moskowitz who has expertise in behavioral interventions further strengthens the team.

Weaknesses

- None.

5. Environment and Institutional Commitment:

Strengths

- Northwestern is an outstanding research institution with many resources available to the candidate.
- Dr. Cella's letter of institutional support addressed concerns about departmental support of the candidate.

Weaknesses

- None.

ADDITIONAL REVIEW CRITERIA (*The following review criteria were not scored individually, but were considered in the determination of the overall impact/priority score*)

Protections for Human Subjects: Acceptable Risks and Adequate Protections

Data and Safety Monitoring Plan: Unacceptable

- A small pilot intervention is planned in Aim 3; however, no DSMP is described.

Inclusion of Women, Minorities and Children:

Both Genders, Acceptable

Minority and Non-minority, Acceptable

Children and Adults, Acceptable

- Participants will be 18+ and the study proposes to recruit patients representative of the RHLCCC population.

Vertebrate Animals: Not Applicable (No Vertebrate Animals)

Biohazards: Not Applicable (No Biohazards)

Resubmission: The candidate and his mentors were very responsive to previous critiques of the career development plan and mentoring. Addition of Aim 3 to pilot and adapt a positive affect intervention significantly enhances the training and research proposed.

ADDITIONAL REVIEW CONSIDERATIONS (*The impact/priority score was not affected by the following considerations*)

Training in the Responsible Conduct of Research: Acceptable

Format: A workshop course for early career faculty who have NIH training grant that incorporates didactic and one-on-one outside discussion with K07 faculty.

Subject Matter: Range of subject matter is appropriate.

Faculty Participation: Mentors are required to engage in discussion of at least 2 topics covered in the course.

Duration: This is a 10 week course (15 contact hours).

Frequency: The course meets weekly for 90 minutes.

Select Agents: Not Applicable (No Select Agents)

Resource Sharing Plans: Not Applicable (No Relevant Resources)

Budget and Period of Support: Recommend as Requested

THE FOLLOWING RESUME SECTIONS WERE PREPARED BY THE SCIENTIFIC REVIEW OFFICER TO SUMMARIZE THE OUTCOME OF DISCUSSIONS OF THE REVIEW COMMITTEE ON THE FOLLOWING ISSUES:

PROTECTION OF HUMAN SUBJECTS (Resume): UNACCEPTABLE
No Data and Safety Monitoring Plan is in place despite the fact that the research plan involves a behavioral clinical trial.

INCLUSION OF WOMEN PLAN (Resume): ACCEPTABLE
Women will be included in the proposed research.

INCLUSION OF MINORITIES PLAN (Resume): ACCEPTABLE
Adequate minority representation is included.

INCLUSION OF CHILDREN PLAN (Resume): ACCEPTABLE
Children over the age of 18 are included.

VERTEBRATE ANIMALS: Not applicable

BIOHAZARDS: No concerns.

TRAINING IN THE RESPONSIBLE CONDUCT OF RESEARCH: ACCEPTABLE

COMMITTEE BUDGET RECOMMENDATIONS: The budget was recommended as requested.

NIH has modified its policy regarding the receipt of resubmissions (amended applications). See Guide Notice NOT-OD-10-080 at http://grants.nih.gov/grants/guide/notice-files/NOT-OD-10-080.html.
The impact/priority score is calculated after discussion of an application by averaging the overall scores (1-9) given by all voting reviewers on the committee and multiplying by 10. The criterion scores are submitted prior to the meeting by the individual reviewers assigned to an application, and are not discussed specifically at the review meeting or calculated into the overall impact score. For details on the review process, see http://grants.nih.gov/grants/peer_review_process.htm#scoring.

MEETING ROSTER

Subcommittee J - Population and Patient-Oriented Training
National Cancer Institute Initial Review Group
NATIONAL CANCER INSTITUTE
NCI-J
June 30, 2011

CHAIRPERSON

ANDRYKOWSKI, MICHAEL A., PHD
UNIVERSITY OF KENTUCKY PROVOST DISTINGUISHED SERVICE PROFESSOR
DEPARTMENT OF BEHAVIORAL SCIENCE
UNIVERSITY OF KENTUCKY COLLEGE OF MEDICINE
LEXINGTON, KY 405360086

MEMBERS

BORGES, VIRGINIA F., MMSC, MD
ASSOCIATE PROFESSOR
DEPARTMENT OF MEDICINE
DIVISION OF MEDICAL ONCOLOGY
UNIVERSITY OF COLORADO DENVER SCHOOL OF MEDICINE
AURORA, CO 80045-253

BOUVET, MICHAEL , MD
PROFESSOR OF SURGERY
CO-DIRECTOR, GASTROINTESTINAL CANCER UNIT
MOORES CANCER CENTER
DIRECTOR OF ENDOCRINE SURGERY
UNIVERSITY OF CALIFORNIA, SAN DIEGO
LA JOLLA, CA 920930987

BROCK, MALCOLM V., MD
ASSOCIATE PROFESSOR OF ONCOLOGY & SURGERY
DIVISION OF CANCER BIOLOGY
JOHNS HOPKINS HOSPITAL
BALTIMORE, MD 21287

CHAO, CHUN , PHD *
RESEARCH SCIENTIST
DEPARTMENT OF RESEARCH AND EVALUATION
SOUTHERN CALIFORNIA PERMANENT MEDICAL GROUP
KAISER PERMANENT
PASADENA, CA 91101

COOLEY, MARY E., RN, PHD
NURSE SCIENTIST
THE PHYLLIS F. CANTOR CENTER
RESEARCH IN NURSING AND PATIENT CARE SERVICES
DANA FARBER CANCER INSTITUTE
BOSTON, MA 02115

HAGENSEE, MICHAEL E., MD, PHD
ASSOCIATE PROFESSOR
DEPARTMENT OF MEDICINE
SECTION OF INFECTIOUS DISEASES
LOUISIANA STATE UNIVERSITY HEALTH SCIENCES CENTER
NEW ORLEANS, LA 70112

HATCHER, JENNIFER , PHD *
ASSISTANT PROFESSOR
COLLEGE OF NURSING
UNIVERSITY OF KENTUCKY
LEXINGTON, KY 40536

HOHL, RAYMOND J., MD, PHD
PROFESSOR OF INTERNAL MEDICINE AND PHARMACOLOGY AND HOLDEN FAMILY CHAIR
ASSOCIATE CHAIR, DEPARTMENT OF INTERNAL MEDICINE
ASSOCIATE DIRECTOR, TRANSLATIONAL RESEARCH
HOLDEN COMPREHENSIVE CANCER CENTER
UNIVERSITY OF IOWA
IOWA CITY, IA 52242

HUDSON, SHAWNA V., PHD
ASSOCIATE PROFESSOR
ROBERT WOOD JOHNSON MEDICAL SCHOOL
DIRECTOR, COMMUNITY RESEARCH
THE CANCER INSTITUTE OF NEW JERSEY
UNIVERSITY OF MEDICINE AND DENTISTRY OF NEW JERSEY
NEW BRUNSWICK, NJ 08903

JACOBSEN, PAUL B., PHD
CHAIR, DEPARTMENT OF HEALTH OUTCOMES AND BEHAVIOR
LEE MOFFITT CANCER CENTER
PROFESSOR, DEPARTMENT OF PSYCHOLOGY
UNIVERSITY OF SOUTH FLORIDA
TAMPA, FL 33612

LAIRD, BEVERLY L. PHD, PHD *
MEMBER
3D MEDICAL CONCEPTS LLC
PELHAM, AL 35124

LAMPE, JOHANNA W., PHD
FULL MEMBER AND ASSOCIATE DIVISION DIRECTOR
CANCER PREVENTION PROGRAM
DIVISION OF PUBLIC HEALTH SCIENCES
RESEARCH PROFESSOR, UNIVERSITY OF WASHINGTON
FRED HUTCHINSON CANCER RESEARCH CENTER
SEATTLE, WA 98109

LOWREY, CHRISTOPHER H., MD
PROFESSOR OF MEDICINE AND PHARMACOLOGY/TOXICOLOGY
DEPARTMENTS OF MEDICINE AND PHARMACOLOGY AND TOXICOLOGY
DARTMOUTH COLLEGE SCHOOL OF MEDICINE
LEBANON, NH 03756

MASKARINEC, GERTRAUD , MD, PHD *
PROFESSOR
EPIDEMIOLOGY PROGRAM
ASSISTANT DIRECTOR FOR RESEARCH TRAINING & EDUCATION
UNIVERSITY OF HAWAII CANCER RESEARCH CENTER
HONOLULU, HI 96813

MONTGOMERY, GUY H., PHD
ASSOCIATE PROFESSOR
DEPARTMENT OF ONCOLOGICAL SCIENCES
DIRECTOR
INTEGRATIVE BEHAVIORAL MEDICINE PROGRAM
MOUNT SINAI SCHOOL OF MEDICINE
NEW YORK, NY 100296574

MOSKOWITZ, CHAYA , PHD *
ASSISTANT ATTENDING BIOSTATISTICIAN
DEPARTMENT OF EPIDEMIOLOGY & BIOSTATISTICS
MEMORIAL SLOAN-KETTERING CANCER CENTER
NEW YORK, NY 10065

MULLINS, C DANIEL, PHD *
PROFESSOR AND CHAIR
PHARMACEUTICAL HEALTH SERVICES RESEARCH DEPARTMENT
SCHOOL OF PHARMACY
UNIVERSITY OF MARYLAND
BALTIMORE, MD 21201

PENEDO, FRANK J., PHD
ASSOCIATE PROFESSOR
DEPARTMENT OF PSYCHOLOGY
UNIVERSITY OF MIAMI
CORAL GABLES, FL 33124

SIU, LILLIAN L., MD
PROFESSOR
UNIVERSITY OF TORONTO
STAFF MEDICAL ONCOLOGIST
DIVISION OF MEDICAL ONCOLOGY/HEMATOLOGY
PRINCESS MARGARET HOSPITAL
TORONTO, ON M5G 2M9
CANADA

SORENSEN, GLORIAN C, PHD *
PROFESSOR
DEPARTMENT OF SOCIETY, HUMAN DEVELOPMENT & HEALTH
HARVARD SCHOOL OF PUBLIC HEALTH
DIRECTOR, CENTER FOR COMMUNITY-BASED RESEARCH
DANA-FARBER CANCER INSTITUTE
BOSTON, MA 02215

WALDMAN, SCOTT A., MD, PHD
SAMUEL MV HAMILTON ENDOWED PROFESSOR
CHAIR, DEPARTMENT OF PHARMACOLOGY AND EXPERIMENTAL THERAPEUTICS
DIRECTOR, DIVISION OF CLINICAL PHARMACOLOGY
THOMAS JEFFERSON UNIVERSITY
PHILADELPHIA, PA 19107

ZAHRBOCK, CARY C., MSW
DIRECTOR
QUALITY IMPROVEMENT AND CANCER SURVIVORSHIP
NATIONAL COALITION FOR CANCER SURVIVORSHIP
UNITED BEHAVIORAL HEALTH - MINNEAPOLIS
MINNEAPOLIS, MN 55418

ZHANG, ZUO-FENG , MD, PHD
DIRECTOR, MOLECULAR EPIDEMIOLOGY & CARCINOGENESIS PROGRAM
UCLA JONSSON COMPREHENSIVE CANCER CENTER
SENIOR PROFESSOR, DEPARTMENT OF EPIDEMIOLOGY
SCHOOL OF PUBLIC HEALTH
UNIVERSITY OF CALIFORNIA AT LOS ANGELES
LOS ANGELES, CA 900951772

SCIENTIFIC REVIEW ADMINISTRATOR

MCKENNA, ILDA M., PHD
SCIENTIFIC REVIEW OFFICER
RESOURCES AND TRAINING REVIEW BRANCH
DIVISION OF EXTRAMURAL ACTIVITIES
NATIONAL CANCER INSTITUTE
NATIONAL INSTITUTES OF HEALTH
BETHESDA, MD 20892

GRANTS TECHNICAL ASSISTANT

HESTER, SHEILA
EXTRAMURAL SUPPORT ASSISTANT
DIVISION OF EXTRAMURAL ACTIVITIES
NATIONAL CANCER INSTITUTE
NATIONAL INSTITUTES OF HEALTH
BETHESDA, MD 208928328

PROGRAM REPRESENTATIVE

LIM, SUSAN E, PHD
PROGRAM DIRECTOR
CANCER TRAINING BRANCH
NATIONAL CANCER INSTITUTE
NATIONAL INSTITUTES OF HEALTH
BETHESDA, MD 20892

MYRICK, DORKINA C, MD, PHD
PROGRAM DIRECTOR
CANCER TRAINING BRANCH
NATIONAL CANCER INSTITUTE
NATIONAL INSTITUTES OF HEALTH
ROCKVILLE, MD 20852

OJEIFO, JOHN O, PHD, MBBS
PROGRAM DIRECTOR
CENTER TO REDUCE CANCER HEALTH DISPARITIES
NATIONAL CANCER INSTITUTE
NATIONAL INSTITUTE OF HEALTH
BETHESDA, MD 20892

PERKINS, SUSAN N., PHD
CANCER TRAINING BRANCH
NATIONAL CANCER INSTITUTE
NATIONAL INSTITUTES OF HEALTH
BETHESDA, MD 20892

ROSEMOND, ERICA K, PHD
PROGRAM DIRECTOR
CENTER FOR CANCER TRAINING
NATIONAL CANCER INSTITUTE
NATIONAL INSTITUTES OF HEALTH
6116 EXECUTIVE BOULEVARD, SUITE 700
ROCKVILLE, MD 20852

* Temporary Member. For grant applications, temporary members may participate in the entire meeting or may review only selected applications as needed.

Consultants are required to absent themselves from the room during the review of any application if their presence would constitute or appear to constitute a conflict of interest.
